# The COVID-19 Cytokine Storm; What We Know So Far

**DOI:** 10.3389/fimmu.2020.01446

**Published:** 2020-06-16

**Authors:** Dina Ragab, Haitham Salah Eldin, Mohamed Taeimah, Rasha Khattab, Ramy Salem

**Affiliations:** ^1^Department of Clinical Pathology, Faculty of Medicine, Ain Shams University, Cairo, Egypt; ^2^Department of Chest Diseases, Faculty of Medicine, Ain Shams University, Cairo, Egypt; ^3^Department of Anesthesia, Intensive Care and Pain Management, Faculty of Medicine, Ain Shams University, Cairo, Egypt; ^4^Department of Radiology, Faculty of Medicine, Ain Shams University, Cairo, Egypt

**Keywords:** COVID-19, cytokine, storm, ARDS, IL-6

## Abstract

COVID-19 is a rapidly spreading global threat that has been declared as a pandemic by the WHO. COVID-19 is transmitted via droplets or direct contact and infects the respiratory tract resulting in pneumonia in most of the cases and acute respiratory distress syndrome (ARDS) in about 15 % of the cases. Mortality in COVID-19 patients has been linked to the presence of the so-called “cytokine storm” induced by the virus. Excessive production of proinflammatory cytokines leads to ARDS aggravation and widespread tissue damage resulting in multi-organ failure and death. Targeting cytokines during the management of COVID-19 patients could improve survival rates and reduce mortality.

## Introduction

In December 2019, an outbreak of pneumonia cases was reported in Wuhan, China. The outbreak was linked to the Huanan food Market. The new virus, 2019-nCoV, so called then, was isolated on 7 January 2020 and identified as the cause of the outbreak (1). The 2019-nCoV virus rapidly spread across China and many other countries and caused a rapidly growing global outbreak. On 11 February 2020, the WHO has named the disease COVID-19, short for “coronavirus disease 2019” (2) and on 12 March 2020 the total number of COVID-19 confirmed cases reached 125,260 globally with 80,981 cases in China and 44,279 outside of China and the COVID-19 was declared to be a pandemic by the WHO (3). As of 26 May 2020, COVID-19 has been confirmed in 5,404,512 individuals globally with deaths reaching 343,514 with a morality of 6.4%, The United States had the highest number of confirmed cases (1,618,757 cases) (4).

## Transmission and Clinical Manifestations of COVID-19

COVID-19 is caused by the SARS-CoV-2 that belongs to the beta-coronaviruses subfamily. Coronaviruses are enveloped, positive single stranded large RNA viruses. Although the first data available about COVID-19 indicates possible animal-to-human transmission via wild animals in Huanan seafood Market in Wuhan (5, 6), epidemiological data and studies, after that, have increasingly demonstrated that the virus transmits human-to-human, through droplets or direct contact, with the reporting that individuals who did not have direct contact with the Huanan seafood market were diagnosed with COVID-19 and with secondary cases occurring at hospitals among health care workers who had extensive contact with COVID-19 patients. The virus was confirmed to spread through respiratory droplets from coughs or sneezes (7–9) with the ability of the host to shed the infection while asymptomatic (10). Studies are now also proposing the possible feco-oral transmission of the virus (11).

COVID-19 patients are mainly adults older than 18 years old with a male predominance, the preconceived notion that pediatrics are not subjected to infection later changed with confirmed cases occurring in pediatrics in China and worldwide (12, 13), however, mortality is still much more in the adult group above the age of 65 years. Adults with pre-existing cardiovascular diseases, respiratory diseases, endocrine diseases, diabetics, or immunocompromised adults remain the most exposed to serious complication of COVID-19 (14).

Although many patients of COVID-19 remain asymptomatic, some patients get pneumonia and 10% of cases require mechanical ventilation and ICU admission. Patients usually present with fever, dry cough, shortness of breath, headache, malaise, muscle, and bony aches. Less common symptoms include sore throat, confusion, productive cough, hemoptysis, diarrhea, nausea, and chest pain (15). Progression to pneumonia is documented by radiological findings and usually occurs 1–2 weeks after the beginning of the symptoms. Signs of pneumonia include decreased oxygen saturation, deterioration of blood gas, multi-focal glass ground opacities, or patchy/segmental consolidation in chest X-ray or CT. Patients presenting late or deteriorating hospitalized patients usually suffer from acute respiratory distress syndrome (ARDS), acute respiratory failure, acute renal injury, and multi-organ failure (15–17).

## Laboratory Findings of COVID-19

Complete blood picture of COVID-19 patients usually shows lymphopenia with or without total leukopenia. A lymphocyte count <1.0 × 10^9^/L has been associated with severe disease (18). A recent research has reported that severe cases of COVID-19 tend to have higher neutrophil to lymphocyte ratio (NLR). NLR is calculated from a routine blood picture by dividing the absolute neutrophil count by the absolute lymphocyte count and indicates a patient's overall inflammatory status. Increasing NLR is a risk factor of mortality not only in infectious diseases but also in malignancy, acute coronary syndrome, intracerebral hemorrhage, polymyositis, and dermatomyostis (19). Platelet count is usually normal or mildly decreased. C-reaction protein and erythrocyte sedimentation rate are usually increased while procalcitonin levels are normal and elevation of procalcitonin usually indicates secondary bacterial infection. Lactate dehydrogenase, ferritin, D-dimer, and creatine kinase elevation is associated with severe disease. Elevation in creatinine or liver enzyme levels (ALT and AST) occurs in complicated cases progressing to multi-organ failure (18).

## Cytokine Profile and The Cytokine Storm

The newly emerging COVID-19 is continuing to challenge medical health systems all over the world and the scenario is still getting worse. The COVID-19 poses an increasing threat to humans with a fatality rate of 6.4 % so far (4). COVID-19 infection is accompanied by an aggressive inflammatory response with the release of a large amount of pro-inflammatory cytokines in an event known as “cytokine storm.” The host immune response to the SARS-CoV-2 virus is hyperactive resulting in an excessive inflammatory reaction. Several studies analyzing cytokine profiles from COVID-19 patients suggested that the cytokine storm correlated directly with lung injury, multi-organ failure, and unfavorable prognosis of severe COVID-19 (16, 20–24).

The immune system has an exquisite mechanism capable of responding to various pathogens. Normal anti-viral immune response requires the activation of the inflammatory pathways of the immune system; however, aberrant or exaggerated response of the host's immune system can cause severe disease if remains uncontrolled (25). Cytokines are an essential part of the inflammatory process. Cytokines are produced by several immune cells including the innate macrophages, dendritic cells, natural killer cells and the adaptive T and B lymphocytes. During an innate immune response to a viral infection, pattern recognition receptors (PRRs) recognize different molecular structures that are characteristic to the invading virus. These molecular structures are referred to as pathogen associated molecular patterns (PAMPs). Binding of PAMPs to PRRs triggers the start of the inflammatory response against the invading virus resulting in the activation of several signaling pathways and subsequently transcription factors which induce the expression of genes responsible for production of several products involved in the host's immune response to the virus, among which are the genes encoding several pro-inflammatory cytokines. The major transcription factors that are activated by PRRs are nuclear factor kB, activation protein 1, interferon response factors three and seven. These transcription factors induce the expression of genes encoding inflammatory cytokines, chemokines and adhesion molecules. This sequence of events results in recruitment of leukocytes and plasma proteins to site of infection where they perform various effector functions that serve to combat the triggering infection (26).

Three of the most important pro-inflammatory cytokines of the innate immune response are IL-1, TNF- α, and IL-6. Tissue macrophages, mast cells, endothelial, and epithelial cells are the major source of these cytokines during innate immune response. The “cytokine storm” results from a sudden acute increase in circulating levels of different pro-inflammatory cytokines including IL-6, IL-1, TNF- α, and interferon. This increase in cytokines results in influx of various immune cells such as macrophages, neutrophils, and T cells from the circulation into the site of infection with destructive effects on human tissue resulting from destabilization of endothelial cell to cell interactions, damage of vascular barrier, capillary damage, diffuse alveolar damage, multiorgan failure, and ultimately death. Lung injury is one consequence of the cytokine storm that can progress into acute lung injury or its more severe form ARDS (27). ARDS leading to low oxygen saturation levels is a major cause of mortality in COVID-19. Although the exact mechanism of ARDS in COVID-19 patients is not fully understood, the excessive production of pro-inflammatory cytokines is considered to be one of the major contributing factors (15–17).

Accumulating evidence suggests that some patients with severe COVID-19 suffer from a “cytokine storm.” Analysis of cytokine levels in plasma of 41 COVID-19 confirmed cases in China revealed elevated levels of IL-1β, IL-7, IL-8, IL-9, IL-10, FGF, G-CSF, GM-CSF, IFN-γ, IP-10, MCP-1, MIP-1A, MIP1-B, PDGF, TNF-α, and VEGF in both patients admitted to the ICU and non-ICU patients compared to healthy adults. All patients included in the study had pneumonia and 1/3 of the patients were admitted to ICU and six of these patients died (16).

A multicenter retrospective study of 150 COVID-19 patients in China evaluated predictors of mortality for COVID-19. The study analyzed data from 82 cases who resolved from COVID-19 and 68 cases who died from COVID-19 and reported significantly higher levels of IL-6 in mortality cases than resolving cases (20). Another study analyzing data from 21 patients in China reported increased levels of IL-10, IL-6, and TNF-α in severe cases (*n* = 11 patients) compared to moderate cases (*n* = 10 patients) (21). A similar study by Gao et al. assessed 43 patients in China and reported that levels of IL-6 were significantly higher in severe cases (*n* = 15) than in mild cases (*n* = 28) (22). Similarly, Chen et al. studied a total of 29 COVID-19 patients, divided into three groups according to relevant diagnostic criteria, and found that IL-6 was higher in critical cases (*n* = 5 patients) than in severe cases (*n* = 9 patients) and that IL-6 was higher in severe cases than in mild cases (*n* = 15 cases) (23).

No much data is available yet regarding severe pediatric COVID-19 patients. A study that evaluated eight critically ill Chinese pediatric COVID-19 patients treated in the ICU, with ages ranging from 2 months to 15 years, reported increased levels of IL-6, IL-10, and IFN-γ among other laboratory findings (24).

Cytokine storm (CS) is a critical life-threating condition requiring intensive care admission and having a quite high mortality. CS is characterized by a clinical presentation of overwhelming systemic inflammation, hyperferritinemia, hemodynamic instability, and multi-organ failure, and if left untreated, it leads to death. The trigger for CS is an uncontrolled immune response resulting in continuous activation and expansion of immune cells, lymphocytes, and macrophages, which produce immense amounts of cytokines, resulting in a cytokine storm. The CS clinical findings are attributed to the action of the proinflammatory cytokines like IL-1, IL-6, IL-18, IFN-γ, and TNF-α (27).

CS has been reported in several viral infections including influenza H5N1 virus (28, 29), influenza H1N1 virus (30), and the two coronaviruses highly related to COVID-19; “SARS-CoV” and “MERS-CoV” (31, 32). Both pro-inflammatory cytokines (e.g., IL-1, IL-6, and TNF-α) and anti-inflammatory cytokines (e.g., IL-10 and IL-1 receptor antagonist) are elevated in the serum of CS patients. The main contributors to the interplay of the cytokine storm are IL-6 and TNF-α. In the absence of an immediate and appropriate therapeutic intervention, patients develop ARDS as a result of acute lung damage followed by multi-organ failure and resulting in death. Hence, the CS should be treated immediately, otherwise mortality can result (28). In addition to anti-viral therapies that can directly target the virus, anti-inflammatory therapies that diminish the cytokine responses are suggested to decrease both the morbidity and mortality in COVID-19 patients.

The early recognition of CS and the prompt treatment can lead to better outcome. Several biological agents targeting cytokines have been proposed for treating CS. IL-1 receptor antagonist, anakinra, which is used in treatment of rheumatoid arthritis, was proven to be helpful in cytophagic histiocytic panniculitis with secondary hemophagocytic lymphohistiocytosis, a disease associated with severe CS (33). Tocilizumab is a recombinant humanized IL-6 receptor antagonist that interferes with IL-6 binding to its receptor and blocks signaling. Tocilizumab is used in treatment of rheumatoid arthritis, juvenile idiopathic arthritis, giant cell arteritis, and has proven valuable in treatment of CS triggered by CAR-T cell therapy for hematological malignancies (34). Downstream inhibitors of cytokines, e.g., JAK inhibitors, are also being explored in treating CS.

As IL-6 is the most frequently reported cytokine to be increased in COVID-19 patients and as IL-6 elevated levels have been associated to higher mortalities, tocilizumab is a candidate drug to be used in managing the cytokine storm accompanying COVID-19. Encouraging results have been reported in China where tocilizumab was used in treatment of 21 patients with severe and critical COVID-19. Clinical data showed that the symptoms, hypoxygenmia, and CT opacity changes were improved immediately after the treatment with tocilizumab in most of the patients, suggesting that tocilizumab could be an efficient therapeutic agent for treatment of the cytokine storm associated with COVID-19 (35). The US Food and Drug Administration (FDA) has approved Roche's Phase III clinical trial of the use of tocilizumab in hospitalized patients with severe COVID-19 pneumonia. The trial is planned to include 330 patients with severe COVID-19 pneumonia (36).

Cytokine storm appears to be one of the common causes of mortality in the recently declared pandemic of COVID-19. Therapeutic approaches to manage the COVID-19 cytokine storm might provide an avenue to decrease the COVID-19 associated morbidity and mortality and is the focus of upcoming studies.

## Author Contributions

All authors contributed to gathering of data, writing, editing, and revising of the manuscript.

## Conflict of Interest

The authors declare that the research was conducted in the absence of any commercial or financial relationships that could be construed as a potential conflict of interest.
